# Expression level of *hTERT* is regulated by somatic mutation and common single nucleotide polymorphism at promoter region in glioblastoma

**DOI:** 10.18632/oncotarget.1975

**Published:** 2014-05-14

**Authors:** Chul-Kee Park, Se-Hoon Lee, Ji Young Kim, Ja Eun Kim, Tae Min Kim, Soon-Tae Lee, Seung Hong Choi, Sung-Hye Park, Il Han Kim

**Affiliations:** ^1^ Department of Neurosurgery, Seoul National University College of Medicine, Seoul National University Hospital, Seoul, Korea; ^2^ Department of Internal Medicine, Seoul National University College of Medicine, Seoul National University Hospital, Seoul, Korea; ^3^ Department of Neurology, Seoul National University College of Medicine, Seoul National University Hospital, Seoul, Korea; ^4^ Department of Radiology, Seoul National University College of Medicine, Seoul National University Hospital, Seoul, Korea; ^5^ Department of Pathology, Seoul National University College of Medicine, Seoul National University Hospital, Seoul, Korea; ^6^ Department of Radiation Oncology, Seoul National University College of Medicine, Seoul National University Hospital, Seoul, Korea; ^7^ Biomedical Research Institute, Seoul National University Hospital, Seoul, Korea; ^8^ Cancer Research Institute, Seoul National University College of Medicine, Seoul, Korea; ^9^ Neuroscience Institute, Seoul National University College of Medicine, Seoul, Korea

**Keywords:** Glioblastoma, hTERT, promoter mutation, hTERT expression, single nucleotide polymorphism, rs2853669

## Abstract

We investigated the role of somatic mutations and a common single nucleotide polymorphism (SNP) in the *hTERT* promoter region on *hTERT* expression and clinical outcomes. The *hTERT* promoter region was sequenced from 48 glioblastomas. *hTERT* expression was analyzed by quantitative real time-PCR. The association between *hTERT* promoter genetic changes and other genomic events and clinical variables common in gliomas were examined. C228T and C250T somatic mutations were found in 60.4% of glioblastomas, and a common SNP (T349C) was found in 66.6%. Somatic mutations and the SNP likely have opposing effects on *hTERT* expression. *hTERT* expression was significantly higher in the C228T or C250T mutated tumors. Tumors with the T349C genotype showed lower *hTERT* expression when C228T or C250T mutations were present. However, no significant survival differences were observed among the groups with or without hTERT promoter mutations and SNP. There was a significant association between genetic changes in the hTERT promoter and patient age as well as MGMT promoter methylation and EGFR amplification. hTERT expression is modulated by somatic mutations in the hTERT promoter as well as a common polymorphism. However, hTERT related genomic changes have limited value as an independent prognostic factor for clinical outcomes in glioblastomas.

## INTRODUCTION

Recent advances in studies regarding non-coding mutations in cancer have inaugurated new chapters for oncogenesis. Of particular importance was the discovery of a *human telomerase reverse transcriptase (hTERT)* promoter mutation in melanoma samples.[1, 2] Researchers found two mutation hotspots, chr5:1,295,228 C>T (C228T) and chr5:1,295,250 C>T (C250T), which can positively regulate *hTERT* transcriptional activity by producing a novel E-twenty-six (Ets) transcription factor binding site.[1, 2] Subsequent studies screening for whole cancer types have discovered that these mutations exist with high frequencies in skin melanoma, thyroid cancer, bladder cancer, hepatocellular carcinoma, squamous cell carcinoma, liposarcoma, and subsets of central nervous tumors, such as glioblastomas, oligodendroglial tumors, and medulloblastomas.[3, 4] Among these, primary glioblastoma has the highest frequency of C228T or C250T hTERT promoter mutations (55%-83%).[3-6]

In the *hTERT* promoter region, there is a single nucleotide polymorphism (SNP), chr5:1,295,349 T>C (rs2853669, T349C), that has been shown to affect telomerase activity and telomere length.[7-9] This functional T349C SNP interferes with Ets2 transcription factor binding and lowers *hTERT* expression in both T/C heterozygotes and C/C homozygotes.[8] A recent study reported that the T349C SNP may even affect the clinical outcome of bladder cancer patients when paired with somatic mutations of C228T or C250T.[10] This common SNP has also been associated with breast cancer risk; however, these reports are controversial.[11-13]

The maintenance of telomere length is essential for cancer cell survival. Telomere lengthening in cancer cells can be explained by either telomerase-dependent or telomerase–independent mechanisms.[14] Considering the high frequency of *hTERT* promoter mutations glioblastomas, it is plausible that the majority of these cells have increased telomerase activity mediated by *hTERT* upregulation. This is strongly supported by recent studies.[3, 5, 15] However, no study has investigated *hTERT* expression in relation to the common SNP in glioblastomas. Furthermore, the reported clinical outcomes associated with *hTERT* expression or *hTERT* promoter mutations in glioblastoma are confusing.[5, 6, 15-18]

We investigated the *hTERT* expression status in glioblastoma samples and its association with clinical outcomes in conjunction with *hTERT* promoter mutations and the common SNP. We also examined the relationship between *hTERT* promoter genetic changes and other representative molecular glioma characteristics, such as *MGMT* promoter methylation, *EGFR* amplification, *IDH1/2* mutation, 1p/19q LOH, and *BRAF* mutation.

## RESULTS

### *hTERT* promoter mutations and common polymorphism

The characteristics of patients whose samples were used in this study are summarized in Table 1. All of the 48 Asian (Korean) patients were histologically diagnosed with glioblastoma. Among them, 29 patients (60.4%) carried the C228T (n=22, 45.8%) or C250T (n=7, 14.6%) somatic mutations in an exclusively. These mutations are predicted to provide a new binding motif (TTCC) for Ets, as described previously (C228T;CTCC→TTCC or C250T; TCCC→TTCC).[2] The other 19 patients (39.4%) carried no somatic mutation in the hTERT promoter region.

**Tabel 1:** Baseline characteristics of the 48 glioblastoma patients

Characteristics	Number of patients	%
Age, years		
≥ 50	28	58.3
< 50	20	41.7
Sex		
Male	28	58.3
Female	20	41.7
Extent of resection		
≥ 95% resection	41	85.4
< 95% resection	7	14.6
Histology		
Glioblastoma	48	100.0
with oligodendroglial component	8	16.7
small cell	1	2.1
rhabdoid	1	2.1
Postoperative performance status, KPS		
≥ 70	43	89.6
< 70	5	10.4
Treatment after surgery		
CCRT/TMZ-TMZ	37	77.1
Hypo-fractionated CCRT/TMZ-TMZ	9	18.7
Others	2	4.2

We identified a common SNP of T349C (chr5:1,295,349 T>C, rs2853669) in the hTERT promoter region in both tumor and blood samples from 32 patients (66.6%). The SNPs existed in both heterozygotes (T/C, n=22, 45.8%) and homozygotes (C/C, n=10, 20.8%). The other 16 patients (33.4%) were T/T homozygotes. The frequency of T349C was higher in this study group compared with those found in the 1000 Genomes database (Table 2).[19] The study population was out of Hardy-Weinberg equilibrium (p=0.00). This T349C SNP is predicted to result in the removal of the Ets2 binding motif (CTTCC→CTCCC).[8]

**Table 2:** The SNP allele frequency (rs2853669, chr5:1259349) in the *hTERT* promoter region with reference to the 1000 Genomes database.[19]

	A/A (T/T)	A/G (T/C)	G/G (C/C)
1000 Genome database			
Genotypes	59.4%	34.1%	6.5%
African, allele fraction (A>G) 0.08	84.6%	14.7%	0.6%
American, allele fraction (A>G) 0.23	59.3%	42.4%	5.3%
European, allele fraction (A>G) 0.29	50.4%	41.2%	8.4%
Asian, allele fraction (A>G) 0.31	47.6%	42.8%	9.6%
Hardy-Weinberg Equilibrium	58.5%	36.0%	5.5%
Present study, 48 glioblastoma patients	33.4%	45.8%	20.8%

**Figure 1:**
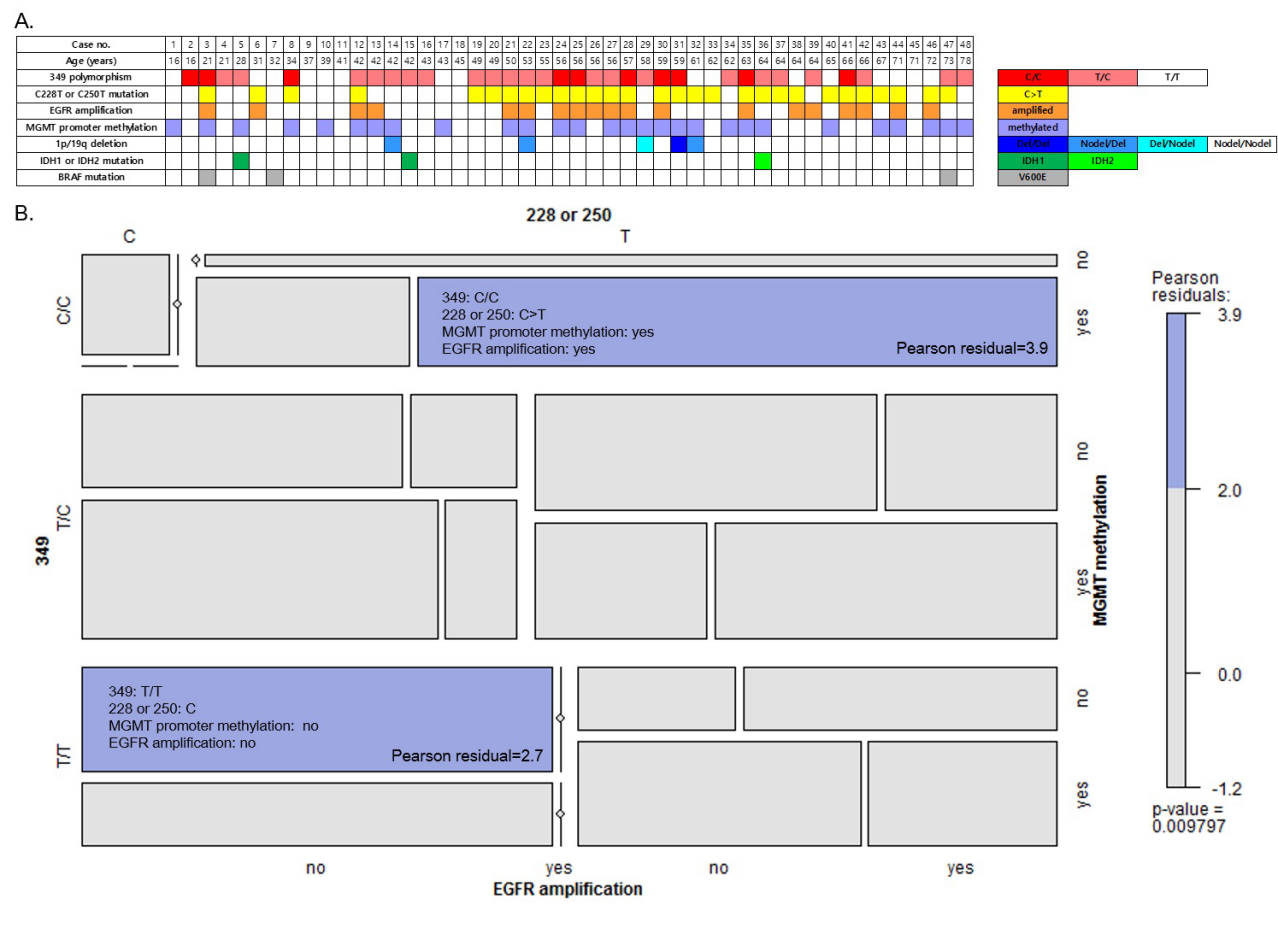
A. Distribution of *hTERT* promoter genetic events and other molecular characteristics in 48 glioblastoma patients. The cases are ordered by priority of age. B. Mosaic plot for somatic mutation (C228T or C250T) and common polymorphism (T349C) in the *hTERT* promoter region, *MGMT* promoter methylation and *EGFR* amplification. The colored cells show significant magnitude of the Pearson residuals (≥ 2.0) obtained from an independence model.

The relationships between genotype and molecular characteristics are summarized in Figure 1A. Considering C228T/C250T and T349C together, 21 patients (43.8%) carried combination of either the C228T or C250T somatic mutation as well as the T349C SNP (T/C or C/C). Eight patients (16.7%) carried the C228T or C250T somatic mutation and the T/T genotype at the 349 SNP site. Among those patients without any somatic mutation in the hTERT promoter, 11 patients (22.9%) carried the T/C or C/C genotypes, and seven patients (14.6%) had the T/T genotype at the 349 SNP site. The mosaic plot shows two significant associations in co-incidence of genotypic and molecular characteristics (Figure 1B). One is T349C (C/C genotype), C228T or C250T somatic mutation, MGMT promoter methylation and EGFR amplification (Pearson residual 3.9). The other is T/T genotype at 349 site with no hTERT somatic mutation, MGMT promoter unmethylation and no EGFR amplification (Pearson residual 2.7). There was no consistent trend of co-incidence between the C228T or C250T somatic mutation and other molecular characteristics, such as chromosomal 1p/19 deletion status, IDH1/2 mutation and BRAF mutation.

**Figure 2:**
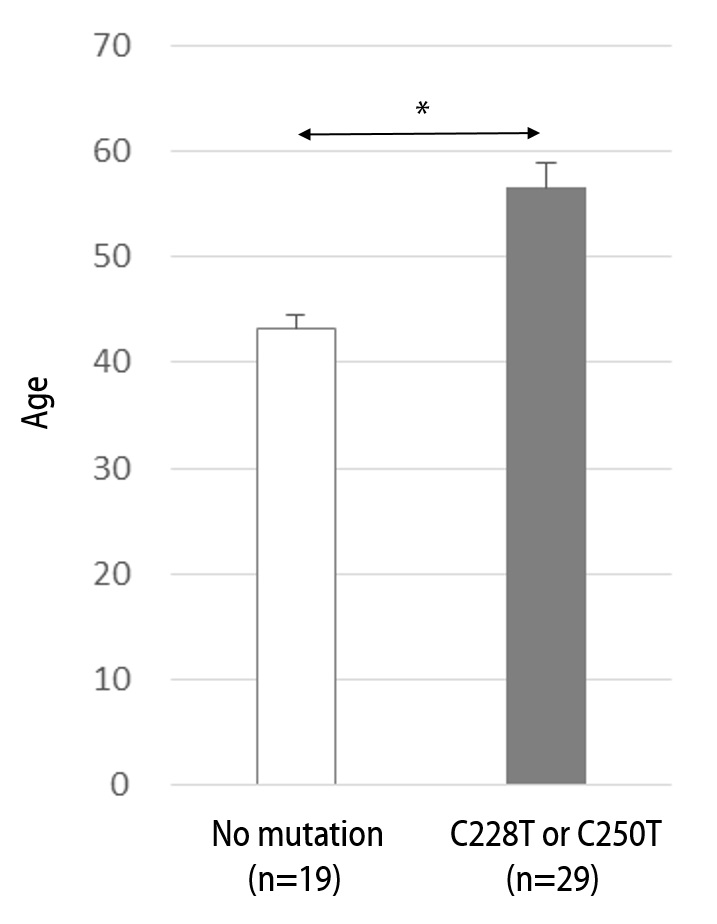
Difference in age distribution according to the hTERT existence of the promoter somatic mutation (C228T or C250T) in glioblastoma patients Data represent mean ± 95% confidential interval. *, p ≤ 0.05.

Among the clinical variables tested, there were significant differences in age distribution between the patients with and without C228T or C250T mutations (Figure 2). Patients with the C228T or C250T mutation were older (mean ± 95% CI, 56.6 ± 4.6 years) than those without the mutation (mean ± 95% CI, 43.2 ± 5.1 years, p=0.00). Other variables were not associated with the *hTERT* somatic mutations, and there were no specific age preponderances according to the *hTERT* common SNP genotypic groups.

### *hTERT* expression

**Figure 3:**
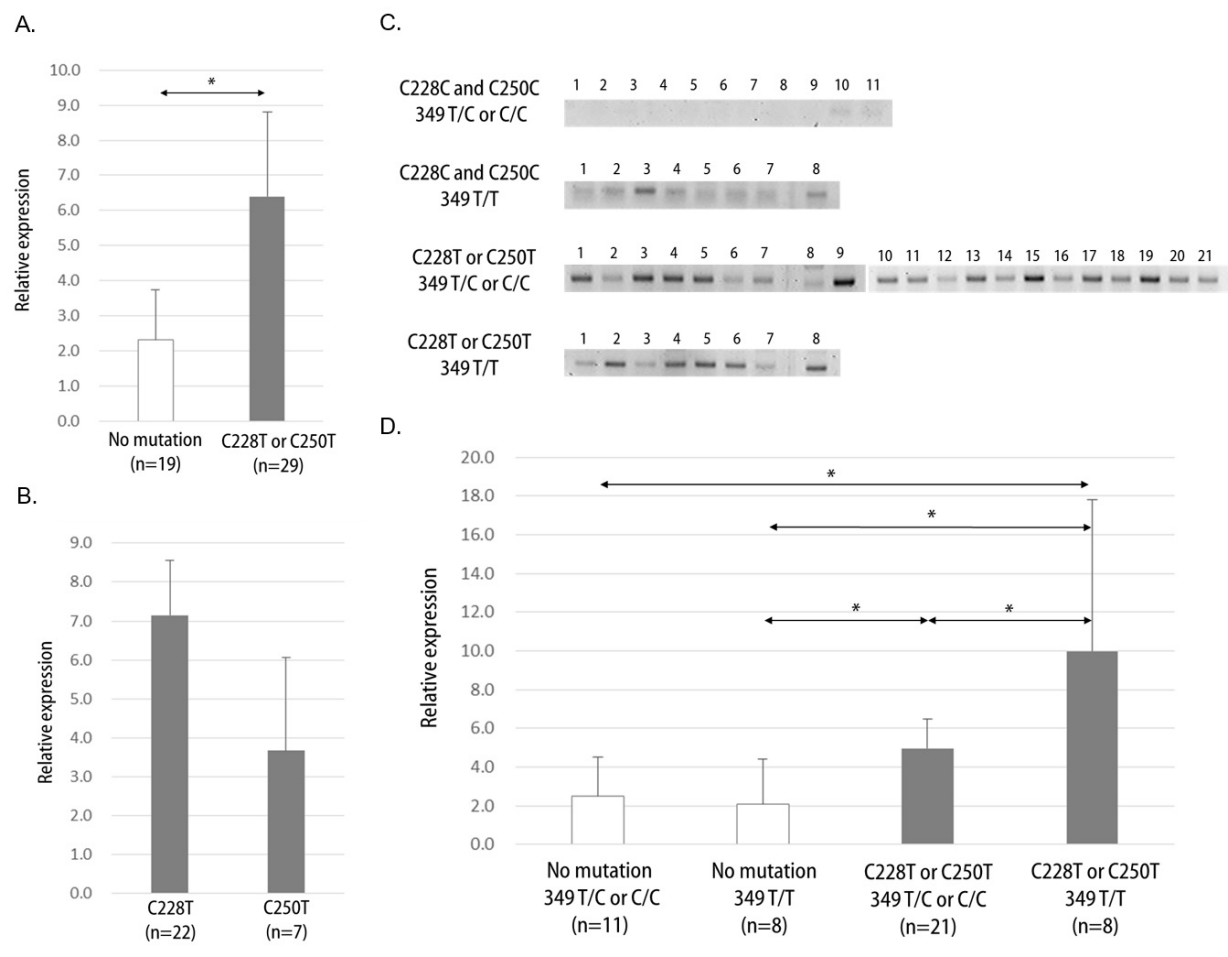
*hTERT* expression in glioblastomas A. Quantitative real time-PCR (Q-PCR) result shows significant *hTERT* overexpression in C228T or C250T mutation samples. B. No differences in *hTERT* expression between C228T and C250T mutation samples were observed by Q-PCR. C. Visualization of reverse transcriptase PCR (RT-PCR) results by groups of *hTERT* promoter genetic events. D. Q-PCR results by groups of *hTERT* promoter genetic events. Data represent mean ± 95% confidential interval. *, p ≤ 0.05.

*hTERT* expression was significantly higher in the C228T or C250T mutation group compared with the no mutation group (Figure 3A, p=0.01). However, no differences in *hTERT* expression were observed between C228T and C250T within the somatic mutation group (Figure 3B, p=0.24). RT-PCR results indicated a modulatory effect on *hTERT* expression in the group with the common T349C SNP (Figure 3C). Quantitative RT-PCR revealed a counterbalance effect on hTERT expression by T349C SNP in C228T or C250T mutated tumors (Figure 3D). Tumors with T349C genotypes (either T/C or C/C) showed lower levels of *hTERT* expression than those with T/T genotype when there was a C228T or C250T mutation (p=0.05). However, the T349C-associated modulation effect was not evident in tumors without *hTERT* promoter mutations.

### Clinical outcome and genetic event of *hTERT* promoter region

**Figure 4:**
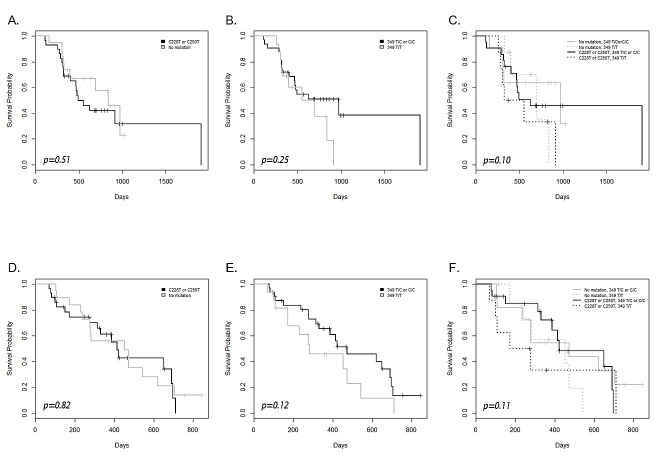
Kaplan–Meier survival plot and the log rank test results for overall survival (A-C) and progression-free survival (D-F) in patients with and without the hTERT promoter mutation, the common polymorphism, and their combination

According to a survival analysis, neither hTERT promoter mutations (C228T or C250T) nor the common SNP (T349C) influenced OS and PFS (Figure 4). Subgroups defined by a combination of C228T or C250T and T349C failed to show an isolated survival outcome (Figure 4). There were also no differences in OS and PFS when the same analysis was done with age-categorized subgroups (data not shown).

## DISCUSSION

The evolution of cancer cells involves genetic and epigenetic changes that enable them to escape natural homeostatic controls. One example is unlimited cellular proliferation and immortality through permanent maintenance of telomere length. In 85-90% of human cancers, this occurs through upregulation of telomerase activity, while in 10-15% of cancers, it occurs through alternative lengthening of telomeres.[14] The *hTERT* gene encodes the catalytic reverse transcriptase subunit of telomerase.[20] Therefore, *hTERT* expression is closely linked to the upregulation of telomerase activity.[15] Because of the rare incidence of somatic mutations in coding region of *hTERT* in cancer cells, genomic events in the promoter region are thought to be the cause of aberrant hTERT expression.[21] The core promoter region of *hTERT* is about 180 bp upstream of the transcription start site and includes GC-rich sequences and multiple transcription factor binding motifs, such as Ets2, SP1, AP2, and c-Myc/Max.[22-24]

The C228T and C250T somatic mutations were the only mutations found in the *hTERT* promoter. These mutations provide an additional binding motif for Ets, which can lead to the upregulation of *hTERT* expression. The high frequency (60.4%) of the C228T and C250T mutations observed in the present study implies that the majority of glioblastomas depend on a telomerase-dependent mechanism for telomere length maintenance. As observed in our data and other studies,[3, 4] there is an obvious preponderance of C228T and C250T mutations in older glioblastoma patients, which provides more evidence for age-specific genetic characteristics in glioblastomas. This suggests that plural mechanisms for gliomagenesis likely exist in glioblastoma. Furthermore, the risk alleles for gliomas found by a genome-wide association study include *hTERT* related SNPs associated with an older age. These findings support distinct pathways of gliomagenesis in those with telomerase associated mechanisms for telomere maintenance.[25]

A high frequency of the common T349C SNP (66.6%) in glioblastoma is another interesting finding. This rs2853669 SNP is also associated with a risk for other cancer types, such as breast cancer and especially lung cancer in the Asian population.[11-13, 26] As summarized in Table 2, the Asian population has the highest A>G allele fraction, and the glioblastoma patients in the present study have significantly lower frequency of the T/T allele and higher T/C and C/C frequency. Considering the lower incidence of glioblastoma in the Korean population (age-standardized rate of 0.59 per 100,000 person-years)[27] compared with that in the United States (age-standardized rate of 3.19 per 100,000 person-years),[28] it would be interesting to determine the frequency of the T349C SNP in glioblastoma patients of other ethnic groups.

Another interesting issue is the evidence in the current study for genomic clustering of the 349 C/C genotype, C228T or C250T somatic mutation, *MGMT* promoter methylation and *EGFR* amplification, as well as of the 349 T/T genotype at the 349 site with no hTERT somatic mutation, and *MGMT* promoter unmethylation and no *EGFR* amplification. The concurrence of *hTERT* promoter mutation and *EGFR* amplification has been confirmed in other studies.[4-6] However, controversial results exist regarding the association between *hTERT* promoter mutation and *MGMT* promoter methylation, IDH1/2 mutation, and 1p/19q deletion in primary glioblastomas.[5] [6] More evidence will be necessary to address this issue more confidently.

Theoretically and actually, a C228T or C250T somatic mutation in the *hTERT* promoter upregulates telomerase expression, as seen in the present study.[3, 5, 29] There is evidence that the T349C SNP is functional polymorphism that can affect *hTERT* expression and even telomerase activity and telomere length.[12] [7] [8] However, as observed in the present study, the major regulator of *hTERT* expression in glioblastoma appears to be a C228T or C250T somatic mutation, though the T349C SNP has modulation effects to some degree. Thus, the role of the T349C SNP may be tissue-specific. A recent study on bladder cancer reported the significance of a T349C SNP for down-regulating *hTERT* expression, which was strong enough to affect survival when coupled with an existing C228T or C250T somatic mutation.[10] On the contrary, the prognostic effect of telomerase activity or *hTERT* expression on survival outcomes in glioblastoma is limited.[15] We could not verify the prognostic effect of either a C228T or C250T somatic mutation or the T349C SNP in the *hTERT* promoter on survival in the present study. Although there is one report showing prognostic significance of *hTERT* promoter mutations in glioblastomas,[4] other studies show no impact on survival by *hTERT* promoter mutations.[5, 6] This evidence implies that *hTERT* is not the major player for therapeutic resistance, although it may be related to gliomagenesis. However, there are evidences that diverse targets for telomerase inhibition can play a role as an anticancer modulator at different levels of cellular process.[30-33] Therefore, further investigation for targeting telomerase is needed to verify the clinical significance of telomerase in cancer.

In conclusion, *hTERT* expression depends on somatic mutations in the *hTERT* promoter, and can also be modulated by a common polymorphism. Genomic changes in the *hTERT* promoter in glioblastoma appear to be associated with age and other representative molecular signatures. However, *hTERT* associated genomic changes appear to have limited value as an independent prognostic factor for glioblastoma clinical outcomes.

## MATERIALS AND METHODS

### Patients and samples

Clinical data and tissue samples from 48 histologically diagnosed glioblastoma patients were collected for the study. Fresh frozen tumor samples and white blood cells were used for DNA and RNA extraction for Sanger sequencing, quantitative real time-PCR (Q-PCR), and reverse transcriptase PCR (RT-PCR). Paraffin-embedded tumor tissues were used for methylation specific PCR (MS-PCR) and fluorescence in situ hybridization (FISH). Manually microdissected tumor areas from 6-μm unstained histologic sections were employed. All the patients were managed with a standard protocol of concomitant radiotherapy with temozolomide as a primary treatment. This study was approved by the local ethics committee, and written informed consent was obtained from all patients.

### DNA and RNA extraction

Genomic DNA was extracted from the frozen tissues and blood using the QIAamp DNA mini kit (Qiagen, Cat. No. 51304), and total RNA was extracted using RNeasy Plus Universal Mini Kit (Qiagen, Valencia, CA, USA, Cat no. 73404). Total RNA was primed with random hexamers to synthesize cDNA using the Quantitect Reverse Transcription Kit (Qiagen, Valencia, CA, USA, Cat no. 205311). DNA was isolated from the paraffin-embedded tissues using a DNeasy Blood and Tissue Kit (Qiagen, Valencia, CA, USA, Cat. No. 69506) according to the manufacturer's instructions.

### Sanger sequencing

The amplification of genomic DNA for the *hTERT* promoter region (size 470bp) was performed using the following primers, forward 5'-ACGAACGTGGCCAGCGGCAG-3' and reverse 5'-CGCGCGTCCCTGCACCCTGG-3'. PCR conditions consisted of an initial heating at 95°C for 5 minutes followed by 35cycles at 95°C for 30seconds, 62°C for 30 seconds, and 72°C for 45 seconds. Amplified PCR products were sequenced using the ABI 3730-XL DNA Sequence Analyzer (Applied Biosystems, Foster City, CA, USA). The sequences were determined using the BigDye Terminator v3.1 cycle sequencing kit (Applied Biosystems, Foster City, CA, USA). To detect BRAF and IDH1/2 mutations, we followed the sequencing procedure described in our previous studies.[34, 35]

### Quantitative real time-PCR and reverse transcriptase PCR

*hTERT* mRNA expression was analyzed by Q-PCR using a TaqMan® Gene Expression Assay (Hs00972650_m1; Applied Biosystems, Carlsbad, CA, USA). Housekeeping gene glyceraldehyde phosphate dehydrogenase (GAPDH) was used for the normalizer. Q-PCR assays were performed twice in triplicate for each sample. The mean *hTERT* level from the U87 cell line was used as the reference to calculate the relative expression using the 2^-ΔΔCт^ method. For RT-PCR, identical primers, amplification conditions, and protocols were used as previously described.[15] The final products were resolved by electrophoresis in 2% agarose gels for visualization.

### Methylation specific PCR

MS-PCR for the purpose of detecting the *MGMT* promoter methylation status was carried out as previously described.[36]

### Fluorescence in situ hybridization

An analysis of 1p, 19q, and *EGFR* gene status was conducted by FISH using Vysis probes and interpreted as described previously.[37, 38]

### Statistical analysis

Pearson contingency analysis was used to analyze mutual correlations of multiple genomic events and was expressed by mosaic plot. Significance of deviance is indicated by Pearson residuals ≥ 2 or ≤ -2 calculated from a chi-square test. Student's t test and ANOVA were used to identify significant differences in distributions of variables and levels of gene expression. Data are presented as the mean ± 95% confidential interval (CI) of three or more separate experiments, and a p value of 0.05 was considered to be the cutoff for statistical significance.

The overall survival (OS) was measured from the date of surgery to the date of the patient's death. Patients who were alive were classified as censored observations at the time of the last follow-up. The progression-free survival (PFS) was defined as a period from the date of surgery to the date of the radiological progression. The Kaplan-Meier method was used to estimate the survival distributions. The log-rank test (level of significance α = 0.05) was used to test the survival differences. All statistical analysis was performed using free software, R (version 3.0.2; http://www.r-project.org/).
